# Social and health epidemiology of immigrants in Germany: past, present and future

**DOI:** 10.1186/s40985-016-0019-2

**Published:** 2016-07-30

**Authors:** Oliver Razum, Judith Wenner

**Affiliations:** Department of Epidemiology and International Public Health, School of Public Health, Bielefeld University, Bielefeld, Germany

**Keywords:** Migration, Germany, Europe, Access, Equity

## Abstract

Germany has experienced different forms of immigration for many decades. At the end of and after the Second World War, refugees, displaced persons and German resettlers constituted the largest immigrant group. In the 1950s, labor migration started, followed by family reunification. There has been a constant migration of refugees and asylum seekers reaching peaks in the early 1990s as well as today. Epidemiological research has increasingly considered the health, and the access to health care, of immigrants and people with migration background. In this narrative review we discuss the current knowledge on health of immigrants in Germany. The paper is based on a selective literature research with a focus on studies using representative data from the health reporting system.

Our review shows that immigrants in Germany do not suffer from different diseases than non-immigrants, but they differ in their risk for certain diseases, in the resources to cope with theses risk and regarding access to treatment. We also identified the need for differentiation within the immigrant population, considering among others social and legal status, country of origin and duration of stay. Though most of the studies acknowledge the need for differentiation, the lack of data currently rules out analyses accounting for the existing diversity and thus a full understanding of health inequalities related to migration to Germany.

## Background

Germany has been an immigration country for decades. At the end of and after World War II, expellees (“*Vertriebene*”) and ethnic German resettlers mainly from Eastern European countries fled or migrated to Germany. During the 1950s the migration of so called guest workers from southern Europe and Turkey started. Later their families followed as part of family reunification. The migration of ethnic German resettlers continued. The right of freedom of movement within the European Union led to an increase of migration to Germany from other EU member states. Since the 1980s, the migration of refugees and asylum seekers increased and now constitutes a large part of migration to Germany [1]. In the political and public discourse, however, Germany was not considered an immigration country. For a long time, immigrants were expected to return to their countries of origin one day, as reflected e.g., in the term guest workers. This changed only after the turn of the millennium. A report published in 2001 by the “Süssmuth-Commission on migration” recognized migration as an important element of the German history since 1945 and also as highly relevant for the country’s future given the demographic transition and the resulting shortage of labor [2]. While there is now consensus that Germany is a major destination country for immigrants, it still does not have a coherent immigration policy. As such it differs from classical immigration countries like the USA and Canada. As a result, approaches towards reception and integration of immigrants differed considerably over time and are still subject to ad-hoc policy changes [3].

The focus of epidemiological research on immigrants’ health also differed between immigrant groups and countries of origin and has changed over time. In the case of guest workers, work-related conditions and culturally determined presentations of symptom were of major interest. Research on refugees and asylum seekers was and is largely concerned with entitlement to health care, communicable diseases and mental health. Since the 1990s, the analysis and explanation of migration-related health inequalities, in particular access barriers, has become a major concern. In the course of this new area of research, topics like diversity management in health care as well as health as a human right are discussed and concepts like the healthy migrant effect and the life-course approach have been developed [4].

The availability of health-related data with information on country of origin, length of stay and socioeconomic status is still a major challenge in epidemiological research on immigrant health in Germany. The term “immigrant” embraces individuals from different groups with regard to region of origin, socioeconomic status and other variables. Hence, there is no “typical” immigrant or even immigrant group – the immigrant population in Germany is heterogeneous. Moreover, immigrants cannot easily be identified in official data bases and health statistics such as cause-of-death statistics (which does not contain information on country of birth). In official data, nationality is available. Foreign nationals can acquire German nationality through naturalization after 8 years of “regular” residence in the country (see also below) and are then no longer identifiable as immigrants in most data bases [5]. In 2005, the concept of “migration background” was introduced in the microcensus (an annual survey of a sample of representative households). Persons with “migration background” either immigrated themselves (the first generation) or are the offspring of first-generation immigrants (the second generation; occasionally, this concept is extended to the third generation) [6].

We use the current debates on health and healthcare of refuges migrating to Germany as an opportunity to look back at previous experiences with immigration to Germany and summarize what is known so far about the determinants of health and the health status of immigrants to Germany. Specifically, we summarize the history of immigration to Germany since the end of World War II and briefly elaborate on the different regulations applying to immigrant groups and their entitlement and access to social services, in particular the health system. We then present an overview of epidemiological research on the health situation of immigrants and point out important determinants of difference in health status between immigrants and non-immigrants in Germany. Finally, we discuss the challenges of tackling health inequalities on the one hand and avoiding the stigmatization of vulnerable groups on the other, with a special focus on the current in-migration of asylum seekers and refugees.

## History of migration and integration

Migration can be defined as “the movement of a person or a group of persons, either across an international border, or within a State” [7]. The former is usually defined as international migration and is the focus of this paper [8]. According to the Population Division of the United Nations’ Department of Economic and Social Affairs, there was a total of 232 million international migrants worldwide in 2013. Germany was the third largest destination country, hosting roughly 10 million international migrants [9]. In research on international migration, destination countries are classified according to the prevailing type of immigration. For a long time after the end of World War II, Germany followed the guest worker model. Immigrants were temporarily welcomed as workforce, but expected to leave the country after 3–5 years. German citizenship was difficult to obtain for persons without German ancestors and integration was not part of the immigration policies. Classical immigration countries like the USA and Canada, but also some European countries (e.g., France and Great Britain) had much more explicit immigration policies and provided pathways to naturalization for regular immigrants [10]. This classification is useful to point out differences between destination countries. At the same time, it simplifies the complex history and present dynamics of migration to Germany, as the following paragraphs show.

At the end of and after World War II, refugees and displaced persons constituted the largest groups of immigrants to Germany. The two German states, the Federal Republic of Germany (West) and the German Democratic Republic (East; both founded in 1949) had to manage the settlement of these millions of persons. The Federal Republic alone received 9.5 million refugees, expellees and ethnic German resettlers until 1981. They were legally considered as Germans at arrival according to Article 116 of the German Basic Law. After 1981 (and especially after the collapse of the Soviet Union), another 3.5 million so called “resettlers” (*Aussiedler* and *Spätaussiedler*) migrated to West Germany from Eastern Europe [1].

In 1955 West Germany ratified the first recruitment agreement with Italy and the migration of guest workers started. Agreements with Spain (1960), Greece (1960), Turkey (1961), Morocco (1963), Portugal (1964), Tunisia (1965), and Yugoslavia (1968) followed. Immigrant workers were supposed to stay in Germany only temporarily and be replaced by new workers regularly. The recruitment took place in the countries of origin and was based on specific selection criteria which considered age, sex, qualification, and also the health status of the workers. The recruitment agreements were just one form of labor migration. Workers from Eastern and Southern European countries also used other ways (such as tourist visa) to migrate to Germany and search for employment themselves [1, 11]. A total number of around 14 million guest workers came to Germany between 1955 and the recruitment stop in 1973. Approximately 11 million returned to their countries of origin in subsequent years. Of those who stayed, the families usually followed under a regulation allowing family reunification during the late 1970s and early 80s after recruitment ended [3]. In the 1980s the number of ethnic German resettlers increased mainly from Poland, the former Soviet Union and Rumania. The 80s also marked the beginning of important migration from asylum seekers and refugees that continued to increase until the mid-1990s [3, 12]. Migration to East Germany was also characterized by work migration during this time period. The majority of foreign workers came from Vietnam and Mozambique. Their absolute numbers were considerably lower than in West Germany [1].

Since the 1990s and still today, two thirds of all immigrants to Germany originate from European Countries of which many are EU member states – at present mainly from Poland, Romania and Bulgaria. Other, numerically smaller immigrant groups today are highly skilled professionals, students, Jewish immigrants from former Soviet Union countries, refugees and an unknown number of undocumented immigrants [3, 13]. At the end of 2014, around 7.2 million foreign nationals lived in Germany, constituting 8.9 % of the total population. The most frequent foreign nationalities were Turkish (approx. 1.5 million), Polish (approx. 670,000) and Italian (approx. 570,000). Of all foreign nationals, 1.3 million (or 1.7 % of the total population) did not migrate themselves, but were born in Germany to parents who migrated or who had a foreign nationality. Another 9.1 million German nationals had a migration background as they migrated to Germany and acquired the German nationality or were born to parents who migrated to Germany. Considering both German nationals and foreign nationals with migration background the number added up to 16.4 million (20.3 % of the total population) in 2014. Individuals with migration background can be grouped according to their (or their parents’) countries of origin: people from the former recruitment countries (mainly from Turkey and Italy) constitute the largest group of 5.9 million (36 %), followed by 4.2 million ethnic German resettlers (mainly from Kazakhstan and the Russian Federation) (26 %) and 2.6 million from member countries of the European Union (16 %). The share of persons with migration background is particularly high among children and persons younger than 20 years (32 %) as well as among young and middle-aged adults (25 % of all adults aged 20 to 45 years) [14, 15].

Germany has been receiving immigrants for many decades. Still, the German integration policy is generally considered to be exclusionist and as such distinguished from more multicultural, assimilatory or pluralist immigration policies [16]. It has, for decades, been characterized by the absence of political efforts to actively foster the societal and political integration of immigrants. Questions of immigration were addressed in executive regulations and not governed by legislative decision. Immigrant workers, refugees and asylum seekers were considered as temporary guests who would – sooner or later – return to their countries of origin. Politically, neither integration nor their naturalization was intended [17]. With regard to naturalization, ethnic German resettlers constitute an exception. They received the German nationality on arrival. The report of the commission on migration published in 2001 is often considered as the turning point in the German immigration policy. Within a few years, the citizenship and naturalization law has been changed, facilitating naturalization and the acquisition of the German nationality for children born to foreign parents with a regular status and at least 8 years of residence in Germany (from *ius sanguinis* to *ius soli*). Finally, a new immigration act was introduced in 2005. It reorganized the immigration law and introduced integration and language courses [3, 18].

Until 1980, less than 50,000 individuals claimed asylum in Germany every year. From the 1980s onwards, the migration of asylum seekers to West Germany (and later on to the reunified Germany) increased and reached its intermediate peak in 1992 during the Yugoslav Wars (1991–2001) with nearly 440,000 asylum claims (mainly by refugees from ex-Yugoslavia). Due to new legislation and the amendment of Article 16 of the German Basic Law in 1993 the number of asylum seekers decreased steadily. The new regulation defined “safe countries of origin” and “safe third countries” from where refugee migration to Germany became nearly legally impossible [19]. In consequence, the number of asylum claims decreased and remained comparatively low until 2012, but increased in some South European countries adjacent to the Mediterranean Sea [20]. In 2015, with the war in Syria and political and economic instability in many other countries, the number reached a new peak with around 477,000 claims. On average, between 30 and 60 % of asylum claims have been rejected in the last decade, putting these asylum seekers at the risk of expulsion [19]. The actual number of refugees and asylum seekers already staying in Germany is supposed to be considerably higher than the number of claims suggests. More than one million have been registered when entering Germany in the last year (2015), but not all of them have claimed asylum so far [21]. The asylum seekers’ countries of origin changes in accordance with global political developments. Half of the newly registered asylum seekers come from Syria, Iraq, Afghanistan and Eritrea [19].

This brief overview of migration to Germany in the past 70 years shows the multitude of countries of origin and reasons for migration as well as the differences in duration of stay. An important conclusion is that immigrants are a heterogeneous population group in many respects. This basic fact is often overlooked when addressing immigrants’ health and health-related needs in epidemiological research.

## Routine data and epidemiological research on migration and health

### Methods and limitations

The most reliable source of representative health data in Germany is the health monitoring system (*Gesundheitsberichterstattung*). Data from official records, administrative records and dedicated surveys is collected and analyzed to picture the health status and the corresponding needs for prevention and care of the population as a whole. Ideally this system should also allow to identify the needs and risk exposures of sub-groups as well as existing health inequalities. Though people with migration background constitute a considerable share of the German population, many data sources used in the official health monitoring system do not routinely include information on the migration status, but only on nationality. So far, only few surveys and records are collecting information on the type of migration, duration of stay, residence status or language skill [4, 22, 23]. In addition to the official health monitoring data, epidemiological studies have been conducted to close this information gap. Mortality and morbidity between immigrants and non-immigrants or between different immigrant groups are compared in order to analyze differences in needs or risks as well as to identify preventable and unjust differences. Ultimately, the research establishes the basis for suitable ways to overcome exiting health inequalities by means of public health interventions, adjustment of health services or strategies concerning other (social) determinants of health [24–27].

The aim of this section is to provide readers with an overview of the health of immigrants in Germany. The selection of the literature included is partly based on the only health report dedicated explicitly to immigrant health that has been published in 2008 and updated with information taken from the current general health report for Germany [5, 23]. Both reports have been published by the Robert Koch-Institute which is responsible for collecting, reporting and communicating health-related information on the federal level in Germany. They form the basis of the review and – wherever necessary – were complemented with research the authors themselves were involved in. A detailed table of health-related data sources and breakdown by migration background in Germany is available here: ([5] p. 26–28).

However, the review remains selective. One the one hand this is due to the authors’ focus on highlighting the possibilities and challenges of epidemiological research on immigrants instead of presenting a comprehensive compilation of data. On the other hand the selectivity is owed to the actual lack of available studies and solid data on health of immigrants.

### Determinants of health of immigrants living in Germany

There are several reasons why the health status and health behavior of immigrants might differ from that of the non-immigrant majority population. In order to understand the reasons for differences in health status, exposures in the pre-migration context in the immigrants’ country of origin, exposures during the migration process and exposures in the post-migration context in the country of destination have to be considered. From a life-course perspective [28], the risks and resources of the immigrants’ pre- and peri-migration context continue to affect their health also after migration, and even in the following generation. This is why the social, economic, environmental, political and cultural context of immigrants before migration needs thorough consideration when the reasons for the prevalence of disease and related risk factors are analyzed [29–32]. The exposures of immigrant workers from a neighboring country like Poland who took two hours by train to cross the border differ substantially from those of war refugees from Syria who lived in damaged cities without proper infrastructure and traveled several months before arriving in Germany. And both differ in risks and resources from German natives who reside in their country of origin, face fewer or no German language problems, and experience neither acculturation stress nor the loss of social networks.

Sometimes genetic or biological differences are identified between immigrant and autochthonous populations. They may lead to differences in the prevalence of diseases, for example diabetes which tends to be more common in immigrant groups originating from South Asia, relative to European populations [33, 34]. These genetic polymorphisms are usually considered as a results of adaptation to environmental conditions. Their role in explaining health inequalities should not be overestimated. First, differences tend to be larger within rather than between population groups [35]. Second, within a population in which a particular genetic polymorphism is common, the usual social gradients operate, with a higher risk of disease among the lower socioeconomic groups. Third, there is growing evidence that disease risk converges over time between immigrant and host populations [36, 37], as would be expected if causes were mainly environmental, rather than hereditary [38, 39].

Besides the explanations related to exposures in the country of origin and the migration process as such (migration-stress-hypothesis), the post-migration context of immigrants living in Germany also differs from non-immigrants [5]. According to Schenk, there are three dimensions of difference that have to be considered in epidemiological research on immigrants: the socio-economic dimension (a), the cultural or ethnic dimension (b) and the legal dimension (c) [40].

- The socioeconomic status of immigrants (income, education and occupation) is on average lower compared to non-immigrants [41]. This is partly due to the migration process and to immigration policies (guest worker model, restrictive recognition of educational qualifications, discrimination and legal restrictions in the access to the labor market and vocational training) and therefore directly linked to the legal dimension [40, 41].
- Cultural differences as well as minority status and related experiences of discrimination have important health consequences. Whereas ethnic communities might be an important source of knowledge and social support, discrimination constitutes a risk for mental illnesses and psychosomatic conditions. In addition, language barriers, low health literacy and lack of diversity-sensitive health services constitute informal barriers to health care and are associated with a risk of inadequate or delayed treatment [42–44].
- Despite the exclusionism that characterized the German immigration policy over decades, regular immigrants and their families – irrespective of their nationality – were (and are) entitled to membership in the statutory health insurance when they are employed or recipients of social welfare. While they may face language and cultural barriers (see b), their entitlement to health care is equal to that of other citizens. This is not the case, however, for asylum seekers and other individuals without regular residence permit (e.g., asylum seekers whose claim was rejected, but whose expulsion is temporarily suspended). For a certain period of time (currently 15 months after arrival) their health expenditure is covered only in case of acute illnesses and pain as well as for pregnancy care and recommended vaccinations. In addition to the limited entitlement they face bureaucratic access barriers as they need to apply for health care vouchers at the local social welfare office before they can see a doctor in most of the federal states [45–47]. Irregular immigrants – without entry or residence permit – have no enforceable entitlement as seeking care might lead to their detection and deportation [48].

Though these multi-dimensional differences suggest that immigrants’ health outcomes may be worse when compared to non-immigrants, empirical studies in Germany (and also in many other destination countries of immigrants) frequently identified an on average lower mortality and morbidity of immigrants. This result also holds true after accounting for lower socio-economic status and adjusting for possible selective return migration [49, 50]. This paradox has been labeled *healthy migrant effect* and has become object of thorough analysis [31, 51, 52]. Several explanations have been proposed: Firstly, immigrants – and especially immigrant workers who form an important part of the immigrant population of Germany – are usually young and healthy at the time of migration. Immigrant workers who followed the official recruitment procedure were screened before migration; persons with disease were not recruited [53]. Thus, immigrant workers are not representative of the population of their country of origin in terms of health. This selection effect may persist for years but subsides when immigrant workers are exposed to work-related risks as well as to other unfavorable social determinants [54]. Secondly, the immigrants’ exposure to risk factors differs between countries of origin and destination. In the case of Germany, most immigrants come from countries with high risk for maternal and infectious causes. They migrate to countries with high risk for chronic and lifestyle-related diseases, but low risk for infectious diseases and good access to health services which prevents maternal causes. However, the risk for lifestyle-related chronic diseases affects immigrants only gradually, whereas the risk for infectious diseases and the better maternal care is reduced rather quickly after migration. Taken as a whole, this may help to explain the lower mortality of immigrants compared to the average German population of the same age [31]. Thirdly, immigrants may possibly emigrate or return-migrate to their country of origin. Not all of them notify the population registries about their migration. The same may be the case for death occurring outside the host country. Thus, the share of foreigners whose death does not materialize in the cause-of-death records (numerator of mortality indicators) though they are still officially registers (denominator of mortality indicators). This may or may not lead to biased mortality rates, but the dimension of the problem is unclear and difficult to measure [30, 49, 50, 54, 55].

The extend of the healthy migrant effect – and more generally of differences in health between immigrants and non-immigrants – varies according to health issue, quality of data, definition of “immigrant”, the selected reference group and the accuracy with which other social, demographic or migration-related determinants can be taken into account. Epidemiological research is slowly trying to account for this heterogeneity within the immigrant population, but the respective data is not always available as we show below.

## Mortality, morbidity and use of health services

### Mortality

Epidemiological studies on mortality in Germany are usually based on cause-of-death records. These records allow for a comparison of mortality rates between German nationals and foreign nationals. Even after standardization for age, the excess mortality of Germans amounted to 37 % for men and 28 % for women [56]. However, the lower mortality of foreigners is not found in all age groups. The mortality of infants born to foreign-born parents, though it has decreased substantially in the last decades, remains higher than that of infants of German-born parents; it seems women with a short duration of residence are at particular risk [5]. Immigrant children up to the age of 19 also experience a higher mortality [56]. The maternal mortality has been high for foreign women in Germany until the late 1990s [57]. Since then, rates do not differ significantly according to nationality [5].

### Non-communicable disease

Cancer risks differ between populations with and without migration background and depend on the type of cancer. Cancer risk among immigrants is related to previous infections which increase the risk of cancer e. g., of the stomach and the liver. Non-immigrants are at higher risk for lifestyle-related cancers such as colon and lung cancer [38]. The cause of death statistics show that non-communicable diseases are the most common cause of death among the population residing (and dying) in Germany. However, a more detailed assessment of the cause of death statistics of 2013 still highlights differences in causes of death according to nationality. Among German nationals, cardiovascular diseases were the most common causes of death while among foreign nationals it were neoplasms [56].

With regard to mental health only few representative studies have been published so far. A review on mental health of immigrants in Germany showed that the reported prevalence of depression, anxiety and posttraumatic stress disorders (PTSD) varies substantially between studies (depression: 3 to 81 %, anxiety: 6 to 90 % and PTSD: 4 to 86 %). Prevalence also varies between migration groups suggesting that refugees and asylum seekers are at higher risk for mental disorders compared to immigrant workers [58]. According to results from the German Health Interview and Examination Survey for Adults (DEGS) the prevalence of symptoms of depression among male immigrants and among first generation female immigrants (those who migrated themselves) is increased compared to non-immigrants (prevalence among women: 9.1 % without migration background, 15.1 % first generation and 14.1 % second generation; prevalence among men: 5.0 % without migration background, 10.6 % first generation and 10.3 % second generation) [59]. However, these findings conflict with studies showing no major difference in prevalence of mental disorders between immigrants and non-immigrants [60]. Given the inconsistency of the results it can be assumed that the prevalence and risk for mental disorders varies considerably within the group of immigrants (e.g., traumatization among refugees and asylum seekers) and between different definitions of disorder as well as the respective methods of inquiry.

Representative data on the health of children and adolescents with and without migration background have become available since the Robert Koch-Institute (RKI) has started the German Health Survey for Children and Adolescents (KiGGS) in 2003. The baseline study enrolled 17,000 children with a representative share of children with migration background. The study identified some differences in health status and health behavior between children and adolescents with two-sided migration background (both parents are immigrants or foreign nationals) compared to children with one-sided migration background and children without migration background. This includes for example the risks for mental illnesses which is higher for children with migration background than for children without migration background. However, this result is partly explainable by the disadvantaged socio-economic status of the families. The analyses also show differences within the group of children with migration background depending on the country of birth (of the parents and the child), the duration of stay and the reason for migration [61, 62].

Data on the prevalence among immigrants of many other non-communicable diseases (e.g., diabetes or coronary heart diseases) is scarce and, where available, possibly not representative [23]. Estimates on the prevalence of diabetes type II among immigrants in Germany, for example, range around 6 % but are difficult to compare to the prevalence in the majority population due to lacking control for age [63].

### Infectious diseases

Data are also lacking regarding the prevalence and incidence of infectious diseases. Only for tuberculosis and HIV/AIDS data stratified by country of origin is available.

As part of the registration of notifiable diseases at the RKI, information about the nationality and the country of birth of individuals with tuberculosis are collected. According to the report on tuberculosis in Germany of 2014, the incidence of tuberculosis among foreign nationals was 33.6 per 100,000 which was 13 times higher than among German nationals (2.5 per 100,000; *p* < 0.001). Of the German nationals with tuberculosis, only 37.6 % were born in Germany. The data suggest that immigrants are at higher risk for tuberculosis as they have been living in in high-risk countries before migration [64]. In Germany, however, transmission mainly occurs within immigrant communities and does not extend to the general population [65].

Reporting of HIV/AIDS is inevitably incomplete as the time of infection and the time of diagnosis might differ considerably. Since 2001, laboratories in Germany are obliged to report positive HIV test results. This allows to estimate the approximate number of people in Germany living with HIV/AIDS. However, the reporting does not include any information about how long and where the respective persons lived after the diagnosis. For 2014, the RKI estimated a total number of 83,400 (95 % KI: 77,000 – 91,200) people living with HIV/AIDS. Of these, 10,400 (12.5 %) had a foreign nationality and have been infected abroad. The majority of HIV-positive persons (87.5 %) had a German nationality and have been infected either in Germany or abroad [66, 67].

Higher prevalence of infectious disease among people with migration background might also be due to an increased risk of infection during visits to their or their parents’ country of origin, as has been shown for Hepatitis A [68].

From the KiGGS study on health of minors we know that the 12-month prevalence of acute ailments (mainly gastrointestinal and respiratory infections) as reported by the parents is lower among children with than without migration background. By contrast, children with migration background are more often only incompletely vaccinated, especially those who were born outside of Germany, and thus at higher risk of vaccine-preventable diseases [61].

### Subjective health

According to the Socio-economic Panel (SOEP) – a longitudinal survey with a representative sample size of around 30,000 participants – the subjective health status of immigrants (from Turkey and other recruitment countries of immigrant workers) is worse compared to non-immigrants of the same age and sex [5]. The subjective health status is a reliable and culturally sensitive indicator for the overall health status and has been identified as a predictor for mortality [69–71]. However, these findings are not controlled for cofounding by socio-economic status which differs on average between immigrants and non-immigrants [5]. In addition, they cannot be confirmed by the DEGS data which did not show major differences in subjective health [59]. This may be due to the cross-sectional character of DEGS. A longitudinal analysis using SOEP data showed that an initially higher health satisfaction of immigrants from Eastern Europe deteriorated over time (i.e., with increasing age and duration of stay) much faster that in the German population, ultimately becoming worse than in the comparison group [72]. Yet an analysis using a different data set showed similar subjective health of Turkish immigrants and Germans after controlling for socioeconomic status and coping resources, in line with the DEGS findings [73].

### Risk factors

In the absence of solid data on the prevalence of chronic diseases like diabetes and coronary heart disease, the distribution of risk factors and risk behavior might provide us with hints at their prevalence. According to the DEGS study, the proportion of smokers among men (first and second generation) and women (only second generation) with migration background is higher than among persons without migration background. For men this difference is mainly due to differences in socio-economic status and does not persist after adjustment [59]. More detailed studies on immigrants’ smoking behavior demonstrate the importance of country of origin, gender, education and duration of stay for the prevalence of smoking among immigrants living in Germany [74, 75]. The analyses of data from the microcensus – a representative annual survey of 1 % of the German population – shows that the prevalence of smoking among female and male ethnic German immigrants from the former Soviet Union slowly converges to the prevalence among the general population of the same sex [74]. This effect was also identified for Turkish immigrants [75].

Microcensus data from 2013 also allows for a comparison of obesity among persons with and without migration background based on self-reported height and weight. For men, no differences in the prevalence of obesity was found whereas the prevalence of obesity was higher in the group of elderly women with migration background than in that without [23]. Again, results are not adjusted for socio-economic status. Moreover, stratification by country of origin and length of stay would be desirable.

Regular physical activity and alcohol consumption do not differ substantially between population groups with and without migration background. Only elderly women (most of whom immigrated long ago) are considerably less likely to be physically active and to show a risky alcohol consumption [59].

With regard to children, a two-sided migration background is associated with a higher prevalence of overweight or obesity (measured) with important differences according to country of origin of the parents. Children with migration background are less physically active and watch television more often. Again this association is largely explained by differences in socio-economic status. On the contrary, the alcohol and tobacco consumption of children with two-sided migration background is lower – especially among children with Turkish or Arabic-Islamic migration background – compared to children without migration background [61].

### Use of health care services

Health systems research has compared the access to, and the utilization as well as the outcome of, health care services between immigrants and non-immigrants in Germany. Entitlements are similar for regular immigrants and the majority population. Restrictions exist for asylum seekers and refugees as well as for the group of irregular immigrants (c.f. section 3.2). There is increasing evidence that these restrictions may contribute to the aggravation of diseases and to an overuse of emergency services [46, 62, 76]. Access barriers are even higher and the health implications potentially more severe for undocumented immigrants [5, 48].

Regular immigrants do not face restrictions in legal entitlements, but there are other access barriers leading to lower utilization and worse outcomes of healthcare. This has been shown for the use of rehabilitative care offered e.g., after cardiovascular disease, cancer treatment, or accidents. Given the growing number of older people with migration background and their often unfavorable working conditions this service is of increasing importance. The analysis showed that foreign nationals use medical rehabilitation less often than the majority population. For those who take part, the objective (occupational performance after the end of rehabilitation) as well as subjective (expressed satisfaction with rehabilitation) outcomes are less satisfactory – even after adjustment for socio-economic, health and demographic characteristics [24, 77]. Findings are similar for the treatment of type-1 diabetes among children and young adults (≤20 years). After adjustment for demographic factors, patients with migration background had worse health outcomes and were less often treated with insulin pump therapy [78, 79]. The DEGS data for adults and the KiGGS study for children show a comparatively low use also of preventive healthcare services and screening among women, men and children with migration background [59, 61].

These observations point at the existence of barriers that immigrants face when accessing health care. In the terminology of Anderson [80], the effective use of health services depends not only on the actual needs, but also on the health system characteristics, predisposing factors (e.g., demographics, social structure, health beliefs) as well as the enabling resources. In the case of immigrants to Germany, barriers with regard to the health system characteristics (e.g., a lack of diversity orientation or other forms of discrimination in health care), the predisposing factors (e.g., low social status and culturally diverging health beliefs) and the lack of enabling resources (e.g., knowledge about the healthcare system, German language proficiency) have been identified [4, 5, 32, 81]. These barriers potentially result in the underuse or inappropriate care with a potentially negative impact on the immigrants’ health.

However, care needs to be taken not to generalize findings. For example, uptake of mammography screening is as high (or even higher) among immigrant women of Turkish origin as in the majority population [82]. Moreover, it appears to be possible to overcome previously existing barriers in the access to adequate health care, as the example of reproductive health in urban areas shows. A study in Berlin did no longer find major differences in the utilization and outcomes of pregnancy-related care between immigrants and non-immigrants. Women with migration background differed neither in the use or outcomes of antenatal care, nor in the frequency of caesarian sections or peri- and neonatal outcomes from women without migration background [83, 84]. Only pregnant women with migration background who lived in Germany for a short time period and who did not master the German language well were more likely to not use antenatal care compared to other women with or without migration background [85].

## Recommendations: considering diversity while avoiding stigmatization

People with migration background – including people with insecure or irregular residence status – will constitute a growing share of the population of Germany in the future [1, 21, 86]. The consideration of their health (care) needs is thus an important public health challenge. Based on our review of the social and health epidemiology of immigrants in Germany, we identify three future priorities for epidemiological research and public health practice.

First, the quality and availability of social and health data for all immigrant groups needs to be improved. The underrepresentation of immigrants in health reporting and epidemiological studies makes it difficult to identify existing heath inequality and health care needs of immigrants. In epidemiological research, the lack of data precludes attempts to picture the diversity of the German population and the heterogeneity within the group of people with migration background [5, 22]. Researcher often have to use broad dichotomized categories like ‘German versus non-German nationality’ or ‘immigrants versus non-immigrants’, with the risk of overshadowing the inter-group differences and neglecting other dimensions of difference, for example the socio-economic status [87]. To actually identify the underlying reasons and mechanisms that lead to health inequalities, detailed assessment of the migration background – including the duration of stay in Germany, the parents’ country of birth, the language spoken at home and the legal status – as well as socio-economic and demographic characteristics is needed. Data availability is even worse for the subgroup of refugees and asylum seekers, though they are among the most vulnerable and fastest growing immigrant groups in Germany. Addressing their health care needs by removing entitlement restrictions is a matter of human rights. Evidence is needed to understand and consider their needs and also to tackle existing inequalities. Epidemiological research as well as the inclusion and identification of refugees and asylum seekers in the official health recording system is thus crucial [22].

Second, there is a need to develop and implement concepts of diversity management in the health care system. Two extremes can be conceptualized when adapting health services to this group – a migrant-specific (“exclusive”) and a migrant-sensitive (“inclusive”) approach. Proponents of a migrant-specific approach argue that health services and prevention programs need to be tailored to the specific needs of immigrants as they differ substantially from those of the majority population. For example, there should be rehabilitation services specifically for immigrants of Turkish origin, with Turkish-speaking staff and Turkish food. A migrant-sensitive approach, however, would consider migration background as one of many dimensions of difference between individuals. Health services should try to cater for the differing needs of all clients, as far as is possible. As a consequence, there is no need for specially tailored programs or services, but rather of a need for openness towards diversity and the increasing heterogeneity of populations [88]. Acknowledging the heterogeneity of the population due to migration experience, religion, language, income, education, gender, age and trying to account for it in the provision of heath care services (and also in research designs) is labeled super-diversity [89, 90]. Further research on how to practically implement this approach is needed.

Third, research on immigrant health has to be self-reflexive and aware of the stigmatizing effects of research results. The example of refugee health highlights the risk of epidemiological studies in minority populations. Research that does not carefully consider the social, structural, and global context of migration and flight might contribute to the stigmatization of immigrants as importers of diseases, burden on the destination country’s health system, and being entirely different in their health risks, needs, and behavior. Epidemiological and public health concerns may thus inadvertently contribute to the social construction of immigrants as being ‘the others’ and as not belonging to society [91]. Such a discourse in turn provokes discriminatory practices in health care, the establishment of obligatory (but not evidence-based) screenings and the restriction of free access to health care for certain groups such as refugees or asylum seekers [76, 92]. Epidemiological research on immigrants’ health – like research on other minority or structurally disadvantaged groups – is thus always on the borderline between the aim to identify and tackle migration-related health inequality on the one hand and the risk of contributing to the stigmatizing discourse and the establishment of discriminatory practices on the other hand. In order to contribute to the former, epidemiological and public health research on migration and health has to reflect on ways to account for super-diversity, needs high quality data that includes detailed information on all dimensions of heterogeneity (or inequality), and has to prevent the misuse of study results for populistic purposes by clearly stating its limitations. The acknowledgment and the realization of the human right to health is at the heart of any approach that aims at the reduction of inequities in health.

## Conclusion

Migration has shaped, and is still shaping, the demographic, social and health situation of the population in Germany. Broadly speaking, immigrants suffer from the same types of diseases as non-immigrants do, but individual disease entities may be more or less frequent, the onset may be earlier or the course more severe. Immigrants may have fewer resources to cope with illness and face barriers towards accessing the required treatment. Differences in prevalence and risk factors or behavior are to a considerable extent the results of social inequalities and the demographic structures of both groups. Thus, migration background is only one dimension of diversity and inequality that interacts with other dimension like socio-economic status or sex/gender. Due to these other social determinants of health, the variance of health status and health literacy within the groups of immigrants is probably just as high as within the population of Germany as a whole, or maybe even higher given the different countries of births and the sustained influence of pre- and peri-migration contexts. In public health research and practice, this diversity as well as the existing migration-related inequalities need to be addressed. Preconditions are the availability of data, the development of diversity-sensitive concepts or services and the avoidance of discriminatory or stigmatizing practices.

## Abbreviations

DEGS, German health interview and examination survey for adults; KiGGS, German health survey for children and adolescents; PTSD, posttraumatic stress disorders; RKI, Robert Koch-Institute; SOEP, socio-economic panel
